# Sex-Based Differences in Lower Extremity Kinematics During Dynamic Jump Landing Tasks After Neuromuscular Fatigue of the Hip Extensors and Knee Flexors

**DOI:** 10.1177/23259671231215848

**Published:** 2023-12-22

**Authors:** Cassidy J.D. Klein, Scott C. Landry, Lauren J. Lattimer

**Affiliations:** † School of Kinesiology, Acadia University, Wolfville, Nova Scotia, Canada; Investigation performed at Acadia University, Wolfville, Nova Scotia, Canada

**Keywords:** biomechanics, electromyography, injury prevention, lower extremity, motion capture

## Abstract

**Background:**

Neuromuscular fatigue can increase the activation of antagonist muscles, thereby reducing the moment produced by the agonist. During the deceleration phase of landing, hip extensor and knee flexor muscles contract eccentrically to counteract the external hip flexion moment. Decreased hip flexion is associated with greater knee extensor moments and risk of injury.

**Purpose:**

To investigate sex-based differences in kinematics and muscle activity after neuromuscular fatigue of the hip extensors and knee flexors during dynamic single-leg tasks.

**Study Design:**

Controlled laboratory study.

**Methods:**

In this study, 9 female (age, 22.3 ± 3.4 years) and 7 male participants (age, 21.3 ± 2.6 years) completed the triple hop (THop) for distance and single-leg drop-jump (SJump) tasks before and after a fatigue protocol consisting of eccentric hip extension and knee flexion. Motion capture and electromyography were used to compare lower extremity kinematics and muscular activation between the sexes.

**Results:**

During the THop, neuromuscular fatigue resulted in significantly decreased maximum hip flexion angles (*P* = .01), maximum knee flexion angles (*P* = .039), and an effect of sex on all hip flexion angles, where both sexes saw decreased hip flexion postfatigue (*P* = .033). A significant interaction of fatigue and sex on hip flexion angular velocity was observed during the SJump, indicating that men experienced an increase while women experienced a decrease in hip flexion angular velocities due to fatigue (*P* = .03). Gluteus maximus activation was increased, and erector spinae activation was decreased postfatigue in women during the THop (*P* = .053 and *P* = .023, respectively).

**Conclusion:**

Results indicate that men and women compensated differently after fatigue of the hip extensors and knee flexors.

**Clinical Relevance:**

Women more commonly assumed an erect landing posture associated with increased injury risk after fatigue of the hip extensors and knee flexors.

Anterior cruciate ligament (ACL) injuries are common in sports. Approximately 70% of these injuries result from noncontact mechanisms and can be attributed to several biomechanical and neuromuscular etiologies.^16,22,37^ Multiple factors are associated with the well-documented sex discrepancy in ACL injuries, where by women are 2 to 8 times more likely to sustain this injury.^2,5,10,20^ The quadriceps and hamstring muscle groups dynamically control the knee, and altered force production of these stabilizers—particularly hamstring deactivation—makes individuals more susceptible to ACL injuries.^29,36^ The quadriceps-to-hamstring ratio compares the concentric hamstring peak torque during flexion with the concentric quadriceps peak torque during extension of the lower limb, and this is a commonly used measure in sports performance and rehabilitation.^13,39^ As the antagonist to the quadriceps, the hamstrings can increase the range of motion at the knee, providing a positional advantage to absorb force more efficiently.^11^ The hamstring muscle group is acknowledged as a synergist to the ACL, as it can assist with resisting anterior translation of the tibia with respect to the femur.^11^ Female participants are considered quadriceps dominant, preferentially activating their quadriceps during dynamic movement more than their male counterparts.^11,26,36^ Increased quadriceps activation in women during landing alters loads at the knee and can increase the risk of injury.^6,15,23,31^ Quadriceps dominance alters external forces generated at the knee by landing in a position with increased knee extension due to a forward position of the individual's center of mass; increases in this external force could then cause the knee joint into a position of further extension. Since the mechanisms from within the body must generate an equal force to this reaction, a torque equal and opposite to the ground reaction is required to protect the athlete upon landing.^11,14^

During dynamic unilateral tasks, limb positioning that increases the risk of injury to the knee includes increased knee valgus, internal hip rotation, and decreased flexion angles at the hip and knee.^11,17,24,27^ Previous literature has indicated that women tend to land with an erect posture in which they lack flexion at the hip and knee joints, inhibiting their ability to adequately absorb impact upon landing and minimize valgus collapse.^11,17,27^ Concentric action of hamstrings during landing allows for greater knee flexion moments and increased knee flexion angles. Eccentric action of the quadriceps and glutes generates knee and hip extension moments that preclude hip and knee flexion, resulting in a more erect landing posture. This highlights that weakness or decreased neuromuscular control of the knee during flexion and extension should be examined when considering mechanisms of ACL injury.^12^

Acute neuromuscular fatigue has been questioned to induce injury to the ACL by reducing the velocity and force of muscle contraction.^15,30^ To further examine the impacts of acute local fatigue, neuromuscular fatigue can be induced in a laboratory setting as a conduit to understanding how and if certain muscle groups contribute to injury risk when weak or fatigued. Neuromuscular fatigue affects athletic performance and the ability to execute sport-specific tasks efficiently.^24,30^ Decreased neuromuscular control alters kinematic variables and proper movement mechanics, which are thought to negatively affect the knee joint, increasing the potential for injury.^24,40^ In particular, these kinematic variables include joint angles such as hip and knee flexion.^1,16,28,37^

When the lower limb becomes fatigued, individuals may exhibit altered motor control, and muscular strength patterns could lead to increased strain on the knee joint.^4,41^ The effects of fatigue have been examined in relation to ACL injury through various interventions, including general and peripheral protocols.^5,20,24,29,32,38,40^ A peripheral fatigue protocol used by Lessi et al^21^ focused on the quadriceps muscle observed that women had greater activation of their biceps femoris postfatigue (POST), and men and women both experienced decreased peak knee flexion angles POST. Liederbach et al^24^ observed different results after a peripheral fatigue protocol of the quadriceps, where participants experienced increased knee flexion angles and decreased knee flexion moments when landing on a single leg. In a general fatigue protocol study, female participants had 22% greater rectus femoris activation than their male counterparts. Both men and women exhibited a significant increase in electromyography (EMG) activation of the rectus femoris upon completion of bilateral drop jumps.^32^ A peripheral fatigue protocol consisting of isokinetic concentric knee flexion movements, observed a reduction in total knee range of motion and knee extensor moments during a counter movement jump POST.^29^ However, in a separate study by Tamura et al,^40^ peak knee flexion angles remained unaltered after a fatigue protocol on a cycle ergometer. In a study examining single-leg drop-jump (SJump) landings, 2 fatigue protocols were administered.^18^ One fatigue protocol focused on maximal concentric effort of the knee extensors, while the second focused on the maximal concentric effort of the knee flexors. The primary findings of this study exhibited that both male and female participants landed with increased knee flexion angles after both fatigue protocols.^18^ However, increased hip flexion angles were seen after the knee extensor fatigue protocol compared with the knee flexor fatigue protocol. This inconsistency within findings suggests that further biomechanical investigations are required to understand how fatigue of certain muscles alters movement patterns, thereby influencing the risk of injury. Specifically, more research into the knee flexors is warranted, as they synergize with the ACL.

Single-leg landings are components of athletic tasks. However, these tasks do not necessarily replicate sports performance. Single-leg landings are singular movements that can be executed outside of athletic settings. It is at the expense of the individual to ensure that the maneuver is being performed adequately. Single-leg landings are used in functional testing, particularly during the triple hop (THop) test. Functional testing may benefit individuals in rehabilitative settings, as the test includes a series of movements with components of acceleration and deceleration on a single leg. Here, individuals can focus on the movement's landing and take-off components. When individuals are completing rehabilitation for their injury, a series of functional tests are used. Functional testing is used to clear an injured athlete for return to play, as these tests put the individual in a vulnerable position but in a controlled setting. In particular, functional jump and hop testing has gained popularity for return-to-play protocols and is commonly used to assess overall functional performance. There is an ongoing debate as to whether functional hop testing is a valid tool for predicting the risk of future injury.^34^ However, it can be used to determine whether one's joint positioning may make them more susceptible to injury. Clinical tests such as the THop are utilized as an appropriate measure of neuromuscular strength, power, and control, as these tests are reliable and easy to administer.^33,34^

There is a gap in the literature for studies specifically focusing on the muscle groups responsible for eccentrically decelerating the lower limb during landings and the accompanying kinematic changes. Examining the specific neuromuscular and biomechanical effects of acute neuromuscular fatigue could highlight how weakness or fatigue of the knee flexors and hip extensors contribute to altered movement patterns that could influence injury risk. This study aimed to investigate hip and knee kinematics and muscular activation patterns after fatigue of the hip extensors and knee flexors and determine whether there are differences between sexes within sagittal plane joint postures during the stance phase of movement while performing the THop for distance and a SJump. We hypothesized that during dynamic THop for distance and SJump landings, women would land with a more erect posture—that is, with decreased hip and knee flexion angles—compared with men POST. A secondary hypothesis was that female participants would experience increased quadriceps activation compared with male participants, who we suspect would experience an increase in hamstring activation to stabilize the knee joint POST. A tertiary hypothesis was generated involving the angular velocities of the hip and knee joint, in which it was hypothesized that men, compared with women, would land with increased knee and hip flexion angular velocities after both single-leg tasks.

## Methods

### Experimental Approach to the Problem

Participants performed dynamic tasks before and after acute bouts of fatigue-inducing muscular contractions to identify the effects of an intervention of neuromuscular fatigue of the knee flexors and hip extensors on lower limb kinematics and muscle activity. Motion capture technology and EMG were used to analyze kinematics and muscle activity during a series of single-leg landing tasks consisting of the THop and SJump to replicate rehabilitative and athletic environments. The THop and SJump data were collected prefatigue (PRE) and POST. The kinematic variables measured about the hip, knee, and ankle included flexion-extension angles and angular velocities. EMG data were collected for the abdominus/internal oblique, vastus lateralis, gluteus maximus, biceps femoris, semitendinosus, and erector spinae. Flexion angles and muscle activation patterns of the surrounding hip and knee joint were measured and analyzed. These tests were used to determine differences in kinematic and muscle activation patterns of the lower limb in an attempt to provide a rationale for identifying alterations in lower limb tasks as a result of neuromuscular fatigue.

### Participants

Study participants were recruited via email and posters at our institution. Potential participants were guided through a face-to-face screening questionnaire before participation to determine eligibility. The exclusion criteria were a history of knee injuries—including ligamental tears and fractures, injuries to the lower limb in the past 6 months, past surgery to the lower limb; and neurological issues—including peripheral nerve damage. A total of 16 participants were included—9 women (age, 22.3 ± 3.4 years) and 7 men (age, 21.3 ± 2.6 years). This was a random sample of participants who were healthy, recreationally active university students, and 2 of the female participants were members of varsity sports teams at the university. Of these participants, 8 women were right-leg dominant, the remaining woman was left-leg dominant, and all men were right-leg dominant (Table 1). Leg dominance was defined as the preferred kicking leg of the participant. Each participant provided written informed consent, and the study protocol received institutional review board approval.

**Table 1:** Demographic Characteristics of Study Participants by Sex^*a*^

Characteristic	Women (n = 9)	Men (n = 7)
Age, y	22.3 ± 3.4	21.3 ± 2.6
Height, cm	169.2 ± 6.9	177.4 ± 8.8
Weight, kg	66.6 ± 14.4	79.1 ± 11.9
Leg dominance, right/left	8/1	7/0

a Data are presented as mean ± SD and n.

### Study Procedures

Participants were asked to attend 1 3-hour session in our motion laboratory. At arrival, participants were instructed to wear tight-fitting and nonreflective clothing. The session involved familiarizing with all apparatus and procedures before conducting the study. Once this was completed, the participant completed the pretesting, fatigue protocol, and posttesting. All testing was completed on both the dominant and nondominant leg. However, data on the dominant leg were analyzed. A Health-o-Meter Professional scale (McCook) was used to determine the height and weight of each participant before beginning the data collection.

#### Electromyography

Participants were equipped with 12 wireless Delsys Trigno EMG sensors (Delsys). The Delsys electrodes recorded raw EMG signals at 2000 Hz. They had preamplifier gains of 1000 times, a bandwidth between 20 and 450 Hz, a common mode rejection ratio of −92 dB, and an input impedance >101^5^Ω. The skin was prepared using alcohol pads and medical-grade razors. Coverlet Cover-Roll Stretch Adhesive Bandages were used to support each sensor. The 12 EMG electrodes were placed bilaterally on the muscles (transverse abdominus/internal oblique, vastus lateralis, gluteus maximus, biceps femoris, semitendinosus, and the longissimus portion of the erector spinae), and electrode placement followed SENIAM (Surface ElectroMyoGraphy for the Non-Invasive Assessment of Muscles) protocols.^9^ A manual muscle testing handheld dynamometer (Microfet 2; Hoggan Scientific) was used for all maximal voluntary contraction (MVC) testing.

Proper MVC techniques were completed using guidelines from previous work (Supplementary Table S1, available separately).^9,19^ When the standardized warm-up was complete, participants completed the MVC portion of the study. The dynamometer was used for the MVC trials (hip extension, medial knee flexion, lateral knee flexion, back extension, knee extension, and abdominal flexion) and measured force in kilogram-force and all MVCs followed the same protocol. Three MVC trials were collected to develop a mean force value produced by the muscle. For the MVC trials, the investigator (C.K.) used standard verbal instructions and instructed participants to reach their maximal contraction in 5 seconds, and verbal encouragement was standardized across all participants.

#### Standardized Warm-up

A dynamic warm-up was completed before the MVC trials and pretesting protocol. The first dynamic warm-up was completed before the MVC trials, and the second dynamic warm-up occurred after the participant was equipped with all retroreflective markers. The first warm-up consisted of participants being instructed to warm up on a stationary bike for 5 minutes. The warm-up also consisted of dynamic exercises—including a standing hip flexor stretch, standing gluteus stretch, lateral lunge, quadriceps stretch, single-leg deadlift, hamstring stretch, and straight-legged walks. After the standardized dynamic warm-up, 3 activation exercises were introduced. A series of 6 bilateral gluteus bridges were completed, followed by 6 repetitions of a cat-and-cow variation and 6 hip hinges. Upon completion of the warm-up exercises, the participant moved on to complete MVCs. The second warm-up consisted of 6 bilateral jumps and 6 hops on both the left and right legs.

#### Motion Capture

A 12-camera motion capture system (Opus 4; Qualisys) and 1 high-speed video camera (Opus 2; Qualisys) captured reflective markers at a sampling rate of 250 Hz. Three AMTI force plates (Advanced Mechanical Technology) captured ground-reaction force and moment data at a sampling rate of 2000 Hz using Qualysis Track Manager software.

A total of 76 retroreflective markers (14 mm) were attached to various anatomic landmarks using double-sided tape. These landmarks included the anterior superior iliac spine (2), posterior superior iliac spine (2), iliac crest (2), jugular notch of the sternum, xiphoid process of the sternum, acromion process (2), T2 spinous process, middle of the inferior angles of the 2 scapulae, medial epicondyle of the humerus (2), lateral epicondyle of the humerus (2), ulnar styloid process (2), radial styloid process (2), posterior triceps (2), 50% of the radial lateral aspect of the forearm (2), medial epicondyle of the femur (2), lateral epicondyle of the femur (2), greater trochanter (2), tibial tuberosity (2), fibular head (2), thigh cluster of 4 markers on the lateral aspect of the thigh (2), shank cluster of 4 markers on the lateral aspect of the calf (2), first distal hallux (2), first metatarsal head (2), second metatarsal head (2), fifth metatarsal head (2), medial calcaneus (2), lateral malleolus (2), medial malleolus (2), posterior heel triad, and last (2) each participant wore a headband with 5 markers attached. Palpation techniques were used to find each landmark. Cramer Colourless Tuf-Skin was used to adhere the markers to the body, and Coverlet Cover-Roll Stretch Adhesive Bandages were used as reinforcement for the markers and to ensure that they remained on the proper landmark for the testing procedure. To secure the retroreflective clusters to the participant, Fabrifoam SupraWraps by Velcro, prewrap, and athletic tape were used.

#### Hop Testing Protocol

**Figure 1.**
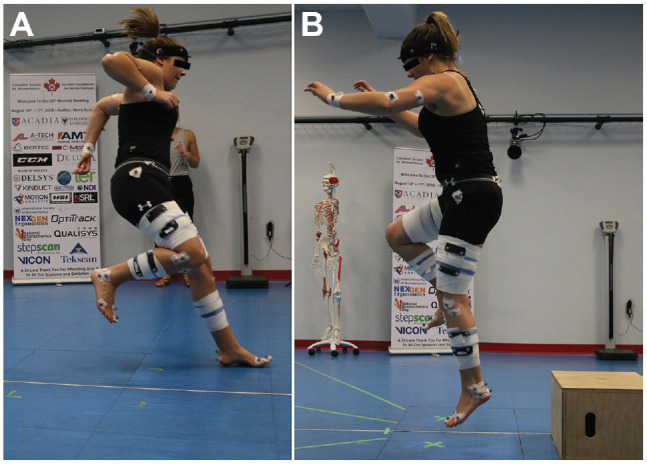
Testing set up for the (A) triple hop and (B) single-leg drop jump.

The THop was adapted from previous literature and was used to assess knee, hip, and ankle joint kinematics PRE and POST.^8,28^ The testing consisted of a timed 6-m hop (T6), a crossover hop (CO), a THop for distance, and a SJump in which a 30-cm wooden box was placed 15 cm from the force plate (Figure 1). A practice trial was given to the participants on each limb being tested. When the practice trial was completed, the 3 recorded trials took place. Participants were instructed to stand with their lead toe behind a predetermined starting line to begin each test. For the jumps and hops to be considered successful, participants were to maintain their balance for 2 seconds. A jump was deemed unsuccessful if the participant touched the ground with the contralateral lower extremity, touched the ground with either upper extremity, was unable to hold their balance, or an extra hop occurred due to being unable to land properly. If an unsuccessful jump/hop occurred, the participant was reinstructed and told to repeat the trial—no further instructions were to be given to the participant. All distance trials were measured to the nearest centimeter, and the timed trials were measured to the nearest thousandth of a second. A tape measure was taped to the floor to regulate the distance covered for all tests. For the SJump, participants were instructed to drop to the opposite foot (dominant), land, perform a SJump, and then land a second time. The participants were able to use their arms for countermovement during the maneuver. Motion capture data were collected during the PRE and POST THop for distance and before and after the SJump. Data for all hop testing, including CO and T6, were collected.

#### Fatigue Protocol

**Figure 2.**
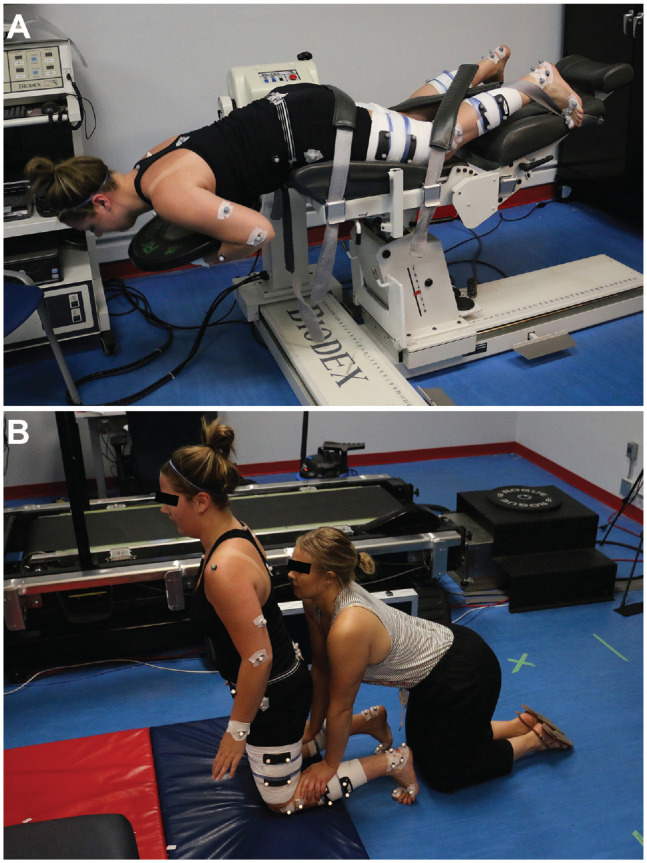
Fatigue protocol consisting of a (A) gluteus hamstring raise and (B) nordic hamstring curl.

The fatigue protocol consisted of gluteus-hamstring raises (GHRs) and nordic hamstring curls. When participants completed the GHRs, they were asked to lay prone on the Biodex System 3 dynamometer (Biodex Medical Systems) with their hips comfortably at the edge of the seat. An 11.34-kg bumper plate was provided to each participant as a standard measure of resistance for the exercises. The participant hinged at their hips so that their torso and head were perpendicular to the floor, and then they picked up the weight and held it to their chest. The participants were instructed to lift their torso to 180° to align with their lower body. They were encouraged to activate their gluteus muscle and their hamstrings at the top of the movement. Standard verbal encouragement was provided during the entire fatigue protocol. The movement was to remain controlled and repeated until the individuals could not successfully complete a repetition. An unsuccessful repetition was defined as being unable to reach 180°. The nordic hamstring curl we used followed the same protocol as a previous study investigating hamstring fatigue in amateur soccer players.^25^ Three sets of maximal effort (weighted) GHRs followed by 3 sets of 15 eccentric nordic hamstring curls were used to fatigue the erector spinae, gluteus maximus, semitendinosus, semimembranosus, and biceps femoris (Figure 2).

When 3 sets of each exercise were completed, a final measurement of fatigue of the medial and lateral hamstrings was taken using the handheld dynamometer, following the same protocol as the MVCs. If the participant's ability to generate force decreased by 20% of the MVC threshold, it was determined that they were adequately fatigued and could proceed to the final set of the fatigue protocol. If they did not fall below the 20% decrease of MVC, the participant repeated 3 more sets of fatigue protocol exercises. To ensure they were fully fatigued before completing the posttesting, a final set of maximum effort GHRs and 15 nordic hamstring curls was completed. Each participant completed a total of 4 rounds of fatigue protocol. The participant immediately started posttesting the dynamic tasks after completing the fatigue protocol. The order in which each dynamic task was performed was randomized.

### Data Analysis

All successfully tracked retroreflective markers, analog force plate data, and EMG data were imported into Visual3D biomechanical software (C-motion). Using a custom-written software program in Matlab R2018b (The Mathworks), the quality of the EMG data was also ensured by carefully observing all waveforms. The 3 trials for each task were averaged, and this software was used to determine the instant contact (IC), maximum (MAX), and mean (MEAN) joint angles during the stance phase. Visual3D was used for all rigid body modeling, and all kinematic data were filtered using a zero-lag, fourth-order, low-pass Butterworth filter with a cutoff frequency of 12 Hz. Three-dimensional joint angles at the hip, knee, and ankle during the THop and SJump were normalized by subtracting the calculated angles during a standing calibration trial. This allowed for joint centers, segment parameters, and reference angles of the joints to be established. Movement calibration trials were performed to identify functional joint centers. Participants performed 3 repetitions of hip flexion/extension, abduction/adduction, and circumduction on each leg while minimizing the torso and hip movement. The raw EMG data from the THop and SJump were full-wave rectified and linear enveloped using a zero-lag fourth-order Butterworth filter with a cutoff frequency of 6 Hz in Visual3D, and then the MVC magnitude was normalized using a 100-ms moving window algorithm for each muscle during each MVC exercise. Muscle activity during the stance phase for the MEAN and the IC was used in the analysis. EMG was further analyzed during the absorption phase for the SJump. The kinematic and EMG data were time normalized so that the stance phase of the THop and SJump was described using 101 data points, with 0% being foot contact and 100% being foot-off.

### Statistical Analyses

Statistical analysis was performed in SPSS 26.0 for Windows 10 Pro (SPSS). Separate group × time repeated-measures multivariate analysis of variance (MANOVA)—2 groups: men and women × 2-time points: PRE and POST—were performed to analyze the hip, knee, and ankle flexion angles and angular velocities at the IC, MAX, and MEAN. A separate group × time (fatigue) repeated measures MANOVA—2 groups: men and women × 2-time points: PRE and POST—was performed to analyze muscle activation of the vastus lateralis, gluteus maximus, biceps femoris, semitendinosus, and erector spinae. Separate MANOVAs for kinematics and muscle activation were performed for both tasks (THop and SJump). A final group × time repeated measures MANOVA (2 groups: men and women × 2-time points: PRE and POST—was used to analyze performance during the THop. If a significant overall multivariate effect or interaction was found, univariate effects were examined for each variable, and Bonferroni-adjusted multiple comparisons testing followed where appropriate. The alpha was set at *P* < .05, and violations of the Mauchly test of sphericity were addressed using a Greenhouse-Geisser correction.

## Results

**Figure 3.**
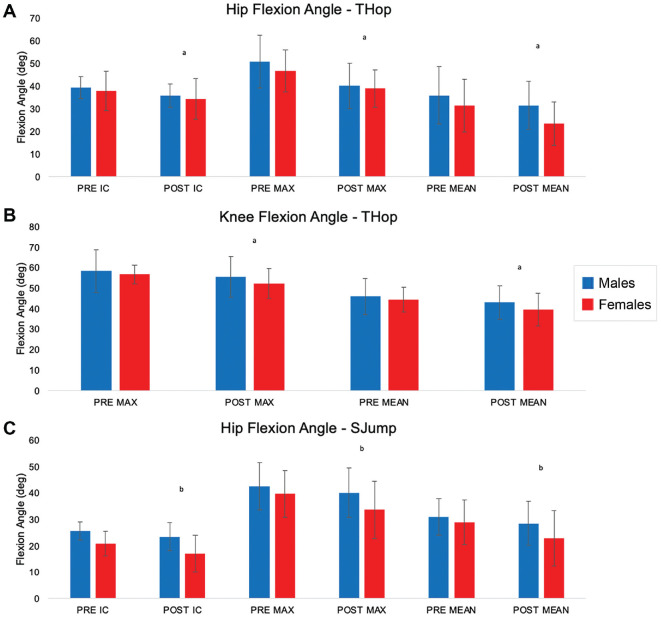
(A) Hip flexion angles for the THop, (B) knee flexion angles for the THop, and (C) hip flexion angles for the SJump stratified by sex at PRE and POST during the IC, MAX, and MEAN. Bars indicate means, with error bars indicating standard deviations. IC, initial contact; MAX, maximum; MEAN, mean; POST, postfatigue; PRE, prefatigued; SJump, single-leg drop-jump; THop, triple hop. ^a^Significant effect of time. ^b^Significant effect of sex.

**Figure 4.**
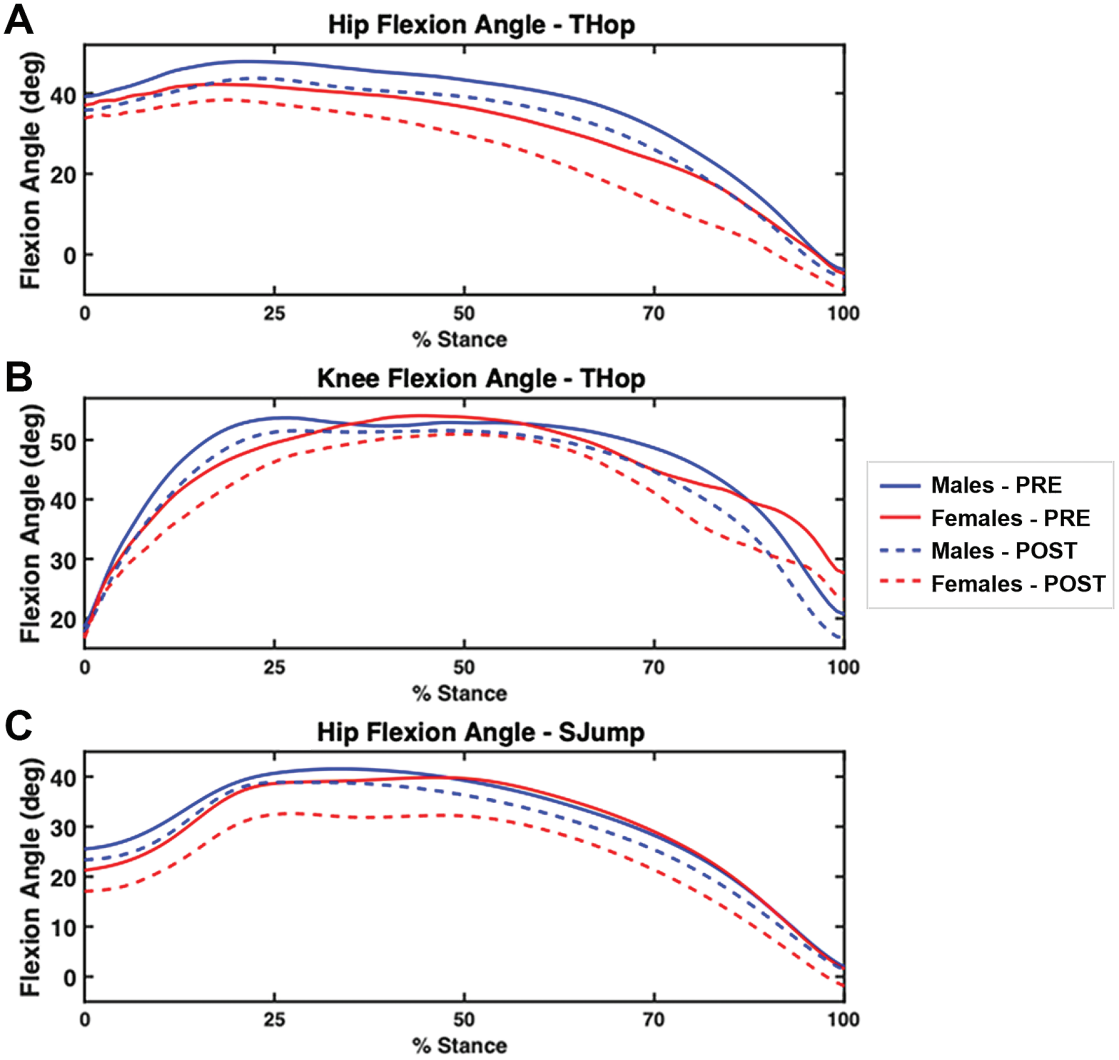
(A) Hip flexion angles for the THop, (B) knee flexion angles for the THop, and (C) hip flexion angles for the SJump stratified by sex at PRE and POST during various points of the stance phase. Mean values shown. POST, postfatigue; PRE, prefatigued; SJump, single-leg drop-jump; THop, triple hop.

For jump distance and timed performance on the THop, CO, and T6 tasks, the multivariate analysis revealed a significant sex effect for all tests (Pillai *T*[3,19] = 4.602; *P* = .014). A significant time effect was found for each test (Pillai *T*[3,19] = 5.528; *P* = .007), showing a decrease in performance after fatigue. The univariate analysis identified a significant time effect for both sexes on the THop (Pillai *T*[1,21] = 17.426; *P* < .001) and T6 (Pillai *T*[1,21] = 6.405; *P* = .19) tasks, indicating that both sexes showed a decrease in performance for POST. The univariate analysis identified a significant sex effect for the THop (Pillai *T*[1,21] = 5.663; *P* = .009) and T6 tasks (Pillai *T*[1,21] = 12.396; *P* = .002), where male participants showed an increase in performance POST, but female participants showed a decrease in performance (distance traveled) POST. The multivariate analysis revealed a significant time effect for hip flexion angles during the THop (Pillai *T*[4,11] = 5.606; *P* = .01), showing that both sexes demonstrated decreased hip flexion angles POST (Figures 3A & 4A). Univariate tests identified a significant time effect for the IC (*F*[1,14] = 10.049; *P* = .007), MAX (*F*[1,14] = 19.284; *P* = .001), and MEAN (*F*[1,14] = 8.011; *P* = .013) hip flexion angles, revealing that both sexes decreased angles POST compared with PRE. The multivariate analysis identified a significant time effect for all knee flexion angle measures (IC, MAX, and MEAN) during the THop (Pillai *T*[3,11] = 3.957; *P* = .039). Univariate tests identified a significant time effect for the MAX (*F*[1,13] = 0.046; *P* = .01) and MEAN (*F*[1,13] = 11.598; *P* = .005) knee flexion angles, indicating that both men and women displayed decreased angles POST (Figures 3B and 4B). During the SJump, a significant sex effect was found for all hip flexion angle measures (IC, MAX, and MEAN) using multivariate analysis (Pillai *T*[3,12] = 4.069; *P* = .033). Univariate analysis found a significant time effect for the IC (*F*[1,14] = 7.249; *P* = .018), MAX (*F*[1,14] = 8.350; *P* = .012), and MEAN (*F*[1,14] = 9.471; *P* = .008) with both men and women experiencing decreased hip flexion angles POST (Figures 3C and 4C).

**Figure 5.**
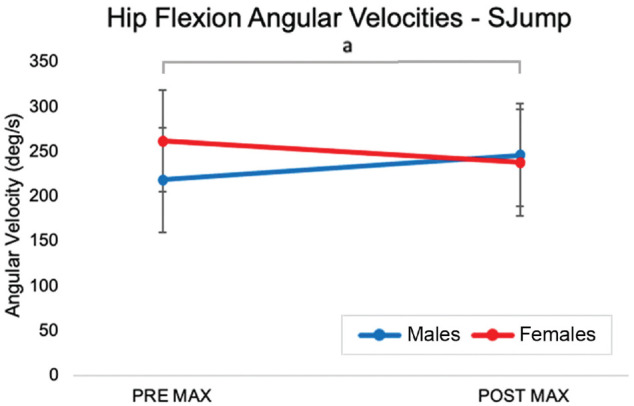
The mean hip angular velocity during the SJump at the MAX during PRE and POST in men and women. Error bars indicate standard deviations. MAX, maximum; POST, postfatigue; PRE, prefatigued; SJump, single-leg drop-jump. ^a^Significant effect of time.

A multivariate analysis revealed a significant sex × time effect for hip angular velocities during the SJump (Pillai *T*[2,13] = 9.235; *P* = .03). Univariate analysis identified a significant sex × time effect for MAX angular velocity (*F*[1,14] = 8.811; *P* = .01), indicating that hip flexion angular velocity increased in men while decreased in women POST (Figure 5).

**Figure 6.**
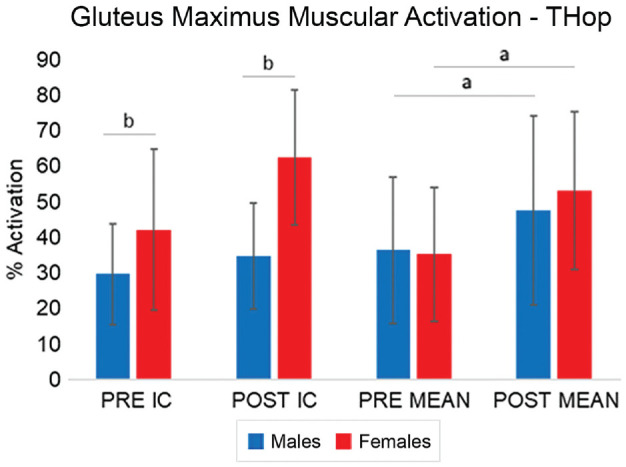
Percentage muscular activation of the gluteus maximus for the THop task PRE and POST during the IC and the MEAN for men and women. Bars indicate means, with error bars indicating standard deviations. IC, initial contact; MEAN, mean; POST, postfatigue; PRE, prefatigued; THop, triple hop. ^a^Significant effect of time. ^b^Significant effect of sex.

The muscle activation analysis showed a significant effect of sex during the THop for the gluteus maximus (Pillai *T*[2,11] = 4.782; *P* = .032). A time effect was also observed (Pillai *T*[2,11] = 5.986; *P* = .017). Univariate analysis revealed a significant time effect on the MEAN muscular activation (Pillai *T*[1,12] = 12.54; *P* = .004), indicating that men and women activated their gluteus maximus to a greater extent after the fatigue protocol. Univariate analysis revealed a significant sex effect on gluteus maximus muscular activation at the IC (Pillai *T*[1,12] = 12.54; *P* = .038), showing that women had greater gluteus maximus activation at the IC (Figure 6).

Multivariate analysis showed a significant sex × time interaction for erector spinae activation during the THop (Pillai *T*[2,13] = 5.083: *P* = .023), and it also showed that the MEAN erector spinae muscle activity increased by 23% in men and 73% in women between PRE and POST.

## Discussion

The hypothesis that women would land in a more erect posture was supported by the present study, as women decreased their hip and knee flexion angles during the THop in a fatigued state. While the main effect of time proved that both sexes decreased flexion at the hip and knee, the decrease was more pronounced in the women. The hip joint angles seemed to be most affected by the reduction in strength of the hip extensors and knee flexors after the fatigue protocol. The difference in hip angle was most prominent in the SJump, with a 20% decrease in hip flexion angles observed in women from PRE to POST, whereas men had a 6% decrease in hip flexion. A study by Thomas et al^41^ expressed similar findings in flexion angles after a THop task. They also reported that women landed with increased hip flexion angles at the IC, which is contradictory to the present study that found women experienced decreased hip flexion. However, the fatigue protocol used by Thomas et al targeted the hamstrings and quadriceps. In the present study, fatigue was focused on the knee flexors, hip extensors, and back extensors. It is possible that as these muscles became fatigued, participants could not effectively control their lower limbs, causing them to land in an extended position, as the knee extensors were not targeted with fatigue. Women are likely to land in this vulnerable and more erect position because they cannot adequately absorb landings, given the decreased ability to produce torque with the hip and back extensors and knee flexors. During the fatigued state, participants compensated by decreasing the MAX and MEAN flexion angle of the hip and the knee to perform the same dynamic tasks. Injury risk could be greater because the compensation pattern of women was more pronounced, resulting in more erect landing than men. The significant changes in joint angles were complemented further by angular velocity to better depict the kinematic changes that occurred in a fatigued state.

Active movement of a joint during landing requires neuromuscular control and aids in energy absorption during landing. Energy absorption during landings can be influenced by angular velocity or resultant joint moments, and greater energy can be absorbed during landings with greater hip and knee angular displacements.^42^ In a fatigue intervention study consisting of an all-women dataset, increased knee flexion angular velocities were observed. However, there was no impact of fatigue on hip flexion angular velocities.^40^ The fatigue intervention utilized in that study was not muscle-specific; participants were required to cycle at 100 watts/min for 5 minutes or until the individual reached a rate of perceived exertion of 17 on the Borg Scale.^40^ The tertiary hypothesis was supported, as the interaction effect identified for hip flexion angular velocities in the present study suggests that in a fatigued state, men may adapt in a positive manner and actively flex their hip through landing, making them more effective in absorbing the impact of the landings. On the other hand, women decreased the active hip movement, which would suggest a stiffer hip posture. The weakness induced by the fatigue protocol caused female participants to resist rapid joint flexion at the hip, while male participants increased flexion velocity at the same level of fatigue. The findings suggest that female participants adjust differently to the same level of fatigue than the male participants, with female participants adopting decreased hip flexion and angular velocity.

Neuromuscular activation of specific muscles allows movements such as flexion and extension, and EMG provides further insight into understanding of the activation of antagonists and agonists that act on a specific joint. Given that women are more likely to land in vulnerable positions that could put them at greater ACL injury risk, a study conducted in 2013 but with no fatigue protocol examined neuromuscular activation patterns during single-leg landings.^35^ Both male and female participants performed a series of SJump landings and replicated an upright landing position, a leaning landing position, and a self-selected landing position. Findings were similar for both sexes, as it was found that there was less lateral quadriceps activation during upright landings compared with leaning landings.^35^ A study that contained a fatigue intervention examined electromyographic changes during bilateral drop jump landings.^32^ Fatigue induced an increase in peak rectus femoris activation by 27%, although there was no statistically significant effect of sex on peak normalized EMG values.^32^ However, it was noted by the authors that women landed with an increased rectus femoris activation of 22% compared with men. This study focused on bilateral landing mechanics, and the present study examined single-leg landing mechanics. It is possible that single-leg landings would elicit similar findings. However, other muscular activation patterns may protect the knee during landing, specifically through activation of the hamstrings and the erector spinae.

We hypothesized that women would increase activation of the quadriceps and that men would increase activation of the hamstrings POST. This hypothesis was rejected, as there were no significant findings related to the hamstrings and quadriceps. Significant findings were found only for the erector spinae and gluteus maximus. In particular, women activated their gluteus maximus greater POST THop than men. At the IC, women had a 67.2% increase in gluteus maximus activation PRE compared with POST, whereas men had a 15.7% increase in gluteus maximus activation. The gluteus maximus is a hip extensor, and it is an antagonist to the hip flexors, meaning that it could put the hip into a more erect posture. In this case, women landed with decreased hip flexion angles during the POST THop testing. At the IC, women also had increased gluteus maximus activity, which suggests that they were recruiting more muscle fibers during landing. Since the recruitment of the gluteus maximus increased, the central nervous system may have compensated by increasing neural input to the muscle due to the presumed loss of muscle strength.^15^ In the fatigued state, male participants increased musculature recruitment of the erector spinae, while female participants did not. To our knowledge, no studies have examined erector spinae activation before and after undergoing a fatigue protocol. A 2016 study by Haddas et al^7^ examined erector spinae activation during SJump landings at 2 different heights. However, no significant differences were seen in activation patterns between sexes. Within the present study, increased erector spinae activity was seen only during the THop task for men, and this increase in activation may be due to the THop requiring more trunk stability during the landings. A possible theory could be that since men were in a more flexed hip position, their erector spinae was in a lengthened position, representing an eccentric contraction that can elicit greater EMG signal. In contrast, women lacked flexion at the hip and possibly relied on the gluteal muscles to control the movement. Interestingly, these findings highlight the importance of investigating a muscle that has not received much attention in the ACL literature.

During the clinical tests (THop, CO, and T6), both men and women showed a decrease in their performance after the fatigue protocol, with women's performance decreasing to a greater extent. The T6 and CO trials were collected for clinical data purposes in which the distance hopped and duration of time were analyzed. Results pertaining to the THop support the hypothesis that both men and women would have a decrease in performance during the clinical trials. After fatigue, both sexes showed a decrease in the distance hopped during the THop and CO. However, only the results for the THop were statistically significant. A statistically significant increase in time was observed in the T6 POST for both men and women. THop testing has been used to assess the performance of the lower extremity, where muscular strength, coordination, and joint positioning are examined.^8^ To our knowledge, no studies have examined the effects of fatigue on THop testing regimes. However, the THop for distance is a predictor of the strength and power of the lower extremity. They found that the THop for distance strongly predicts quadriceps and hamstring muscle strength. Based on these findings and understanding of how muscle fatigue causes reductions in the ability to perform sport-specific tasks involving balance, control, and joint stability,^3,5,24,30^ it can be assumed that fatigue caused reductions in performance during THop testing, as the hop distance was decreased and the time increased during the T6. This suggests that fatigue did affect the participant's ability to execute the tasks, most likely due to the lack of motor control and decreased strength and power. We conclude that this battery of tests can be used to detect fatigue, which could be used to assess an athlete's motor control, strength, and power before and after a training intervention.

Participants in the present study experienced various changes in joint kinematics and muscle activation that contribute to joint postures and could make one susceptible to injury. This study highlights compensation patterns that women exhibit when they have weakened or fatigued hip extensors and knee flexors. To our knowledge, this is the first study of its kind to examine the effects of fatigue of the decelerators of the lower limb on single-leg landing tasks, including the THop for distance and the SJump. We observed that specific fatigue of the muscles, which eccentrically control single-leg landing, resulted in sex-related kinematic changes that could influence ACL injury risk. The finding related to hip flexion angular velocities suggests that men and women actively adopt different changes in joint angles that may contribute to landing in vulnerable positions. Our EMG data analysis indicated that men and women adopt differing muscle activation patterns when the torque-producing capabilities of the posterior chain muscles are decreased, suggesting that men and women have different protective mechanisms in navigating fatigue of these muscles. Findings from the present study provide rationale that fatigue potentially influences ACL injury risk and may provide insight into developing specific strength training interventions and rehabilitative measures, which attempt to mitigate the risk of noncontact ACL injuries.

### Limitations

The present study did identify significant findings supporting that specific fatiguing of the muscles that eccentrically control landing may, in fact, lead to an increased risk of ACL injury. However, there were some limitations of the study. First, the present study had a small sample size. Had the sample size been larger, more significant interaction effects regarding how men and women adapt to fatigue differently may have been identified, specifically regarding muscular activation patterns, which could provide further insight into why specific joint angles and angular velocities were observed. Second, using an intense peripheral fatigue protocol may not be directly applicable to the more global fatigue that occurs in sports. The findings of this study can only be generalized to a random sample of recreationally active individuals with no considerations of training background or status.

## Conclusion

The participants in the present study experienced various changes in joint kinematics and muscle activation that contribute to joint postures that could make one susceptible to injury, and there were differences between the sexes. We observed that peripheral fatigue resulted in sex-related changes in kinematics that are related to ACL injury risk. Overall, after the fatigue protocol, participants reduced flexion at the hip and the knee during the THop, but male participants experienced greater knee flexion than female participants. After fatigue, men flexed their hips more and had increased hip angular velocity compared with women during the SJump. These findings indicate that a similar fatigue protocol resulted in differing kinematic changes between sexes. Muscle activation patterns also differed between sexes, where women experienced greater gluteus maximus activation after fatigue and men increased recruitment of their lower back musculature.

## Supplemental Material

sj-pdf-1-ojs-10.1177_23259671231215848 – Supplemental material for Sex-Based Differences in Lower Extremity Kinematics During Dynamic Jump Landing Tasks After Neuromuscular Fatigue of the Hip Extensors and Knee Flexors Click here for additional data file. Supplemental material, sj-pdf-1-ojs-10.1177_23259671231215848 for Sex-Based Differences in Lower Extremity Kinematics During Dynamic Jump Landing Tasks After Neuromuscular Fatigue of the Hip Extensors and Knee Flexors by Cassidy J.D. Klein, Scott C. Landry and Lauren J. Lattimer in Orthopaedic Journal of Sports Medicine
