# Supplementary material for: Sex-Based Differences in Lower Extremity Kinematics During Dynamic Jump Landing Tasks After Neuromuscular Fatigue of the Hip Extensors and Knee Flexors
Source: Orthop J Sports Med. 2023 Dec 22;11(12):23259671231215848. doi: 10.1177/23259671231215848 (PMC10748927; doi:10.1177/23259671231215848)
Supplement: sj-pdf-1-ojs-10.1177_23259671231215848 – Supplemental material for Sex-Based Differences in Lower Extremity Kinematics During Dynamic Jump Landing Tasks After Neuromuscular Fatigue of the Hip Extensors and Knee Flexors [file sj-pdf-1-ojs-10.1177_23259671231215848.pdf]

Supplemental Table S1. Guidelines for MVC Techniques

Muscle	Placement	Activation	MVC
**Transverse Abdominus/Internal Oblique**	Participants laid in supine position with their knees bent to approximately 90°. The anterior superior iliac spine (ASIS) was palpated, the electrode was placed horizontally 2 cm inferomedial to the ASIS	Participants were instructed to hollow out their abdomen by drawing their naval up and in towards the spine.	Participants were instructed to draw their naval up and in towards the spine, hollowing their abdomen.
**Vastus Lateralis**	Participants laid in a supine position with the knees slightly flexed. The electrode was placed 2/3 on the line from the ASIS to the lateral side of the patella, orientated in the direction that the muscle fibers run.	Participants were instructed to push the posterior aspect of their knee into the plinth to test placement.	Participants were instructed to sit with knee bent and hanging over edge of the table. The HHD was placed above the ankle. The participant was asked to extend the knee with maximal strength. The participant’s arms were to be crossed over their chest.
**Gluteus Maximus**	Participants laid in a prone position. The electrode was placed 50% on the line between the sacrum and the greater trochanter. The electrode was orientated to follow the direction of the muscle fibers	Participants were instructed to lift their entire leg from the table, performing hip extension to test placement.	Participants were instructed to lie prone with knees extended. The HHD applied resistance to the distal thigh to stimulate hip extension.
**Biceps Femoris**	Participants laid in a prone position on a plinth. The electrode was placed 50% from the ischial tuberosity to the lateral epicondyle of the tibia. The electrode was placed vertically on the leg in line with the direction of the muscle fibers.	Participants were instructed to resist knee flexion to test electrode placement.	Participants were instructed to lie prone with the knees bent to 90 degrees. The HHD was placed at the calcaneus. The participants were instructed to draw their calcaneus to the lateral portion of their gluteus maximus to activate the lateral hamstring.
**Semitendinosus**	Participants laid in a prone position on a plinth. The electrode was placed 50% from the ischial tuberosity to the medial epicondyle of the tibia. The electrode was placed vertically on the leg in line with the direction of the muscle fibers	Participants were instructed to resist knee flexion to test electrode placement.	Participants were instructed to lie prone with the knees bent to 90°. The HHD was placed at the calcaneus. The participants were instructed to draw their calcaneus to the medial portion of their gluteus maximus to activate the medial hamstring.
**Erector Spinae (Longissimus)**	Participants laid in a prone position on a plinth. The electrode was placed 2 finger widths lateral of the spinous process of the L1 vertebrae. The electrode was placed in a vertical position, following the muscle fibers.	Participants were instructed to lift trunk in a prone position to test muscle placement.	Participants laid prone, with hips at the edge of the Biodex machine. Their lower body was strapped in to maintain stability. The HHD was placed between the inferior angles of the participant’s scapulae in a downwards motion activating the back extensors.

From Lattimer LJ, Lanovaz JL, Farthing JP, Madill S, Kim S, Arnold C. Upper limb and trunk muscle activation during an unexpected descent on the outstretched hands in young and older women. *J Electromyogr Kinesiol*. 2016;30:231-237.
